# Causal association between the peripheral immunity and the risk and disease severity of multiple sclerosis

**DOI:** 10.3389/fimmu.2024.1325938

**Published:** 2024-02-08

**Authors:** Lian Chen, Li-Fang Zhu, Lu-Yang Zhang, Yun-Hui Chu, Ming-Hao Dong, Xiao-Wei Pang, Sheng Yang, Luo-Qi Zhou, Ke Shang, Jun Xiao, Wei Wang, Chuan Qin, Dai-Shi Tian

**Affiliations:** ^1^Department of Neurology, Tongji Hospital, Tongji Medical College, Huazhong University of Science and Technology, Wuhan, China; ^2^Hubei Key Laboratory of Neural Injury and Functional Reconstruction, Huazhong University of Science and Technology, Wuhan, China

**Keywords:** multiple sclerosis, Mendelian randomization (MR), genome wide association study (GWAS), peripheral immune cells, cytokines

## Abstract

**Background:**

Growing evidence links immunological responses to Multiple sclerosis (MS), but specific immune factors are still unclear.

**Methods:**

Mendelian randomization (MR) was performed to investigate the association between peripheral hematological traits, MS risk, and its severity. Then, further subgroup analysis of immune counts and circulating cytokines and growth factors were performed.

**Results:**

MR revealed higher white blood cell count (OR [95%CI] = 1.26 [1.10,1.44], P = 1.12E-03, P adjust = 3.35E-03) and lymphocyte count (OR [95%CI] = 1.31 [1.15,1.50], P = 5.37E-05, P adjust = 3.22E-04) increased the risk of MS. In further analysis, higher T cell absolute count (OR [95%CI] = 2.04 [1.36,3.08], P = 6.37E-04, P adjust = 2.19E-02) and CD4^+^ T cell absolute count (OR [95%CI] = 2.11 [1.37,3.24], P = 6.37E-04, P adjust = 2.19E-02), could increase MS risk. While increasing CD25^++^CD4^+^ T cell absolute count (OR [95%CI] = 0.75 [0.66,0.86], P = 2.12E-05, P adjust = 1.72E-03), CD25^++^CD4^+^ T cell in T cell (OR [95%CI] = 0.79[0.70,0.89], P = 8.54E-05, P adjust = 5.29E-03), CD25^++^CD4^+^ T cell in CD4^+^ T cell (OR [95%CI] = 0.80[0.72,0.89], P = 1.85E-05, P adjust = 1.72E-03), and CD25^++^CD8^+^ T cell in T cell (OR [95%CI] = 0.68[0.57,0.81], P = 2.22E-05, P adjust = 1.72E-03), were proved to be causally defensive for MS. For the disease severity, the suggestive association between some traits related to CD4^+^ T cell, Tregs and MS severity were demonstrated. Moreover, elevated levels of IL-2Ra had a detrimental effect on the risk of MS (OR [95%CI] = 1.22 [1.12,1.32], P = 3.20E-06, P adjust = 1.34E-04).

**Conclusions:**

This study demonstrated a genetically predicted causal relationship between elevated peripheral immune cell counts and MS. Subgroup analysis revealed a specific contribution of peripheral immune cells, holding potential for further investigations into the underlying mechanisms of MS and its severity.

## 1 Introduction

Multiple sclerosis (MS) is one of the most common chronic inflammatory demyelinated disorders affecting the central nervous system (1). It frequently leads to high rates of disability and recurrent episodes among young people, causing impairments in sensation, mobility, and cognitive function (2). Currently, MS affects 2.8 million people worldwide and is diagnosed in one person at an average age of 32 every five minutes (3). Unfortunately, the available evidence is insufficient to establish the effectiveness of drug treatment in halting or reversing the progression of MS, primarily due to the limited understanding of the underlying mechanisms driving its pathogenesis.

Immunological mechanisms play a crucial role in the progression of MS, leading to neurological damage. Aberrant activation of lymphocytes, specifically CD8^+^ and CD4^+^ T cells, contributes to the destruction of oligodendrocytes and neurons in active MS lesions (4). Additionally, MS patients often exhibit increased peripheral blood Th17 cells and elevated levels of Th17-related cytokines in their serum (5). B cells are also involved in the formation of lesions throughout various stages of the disease, as indicated by the presence of oligoclonal bands in MS (6). Given these findings, many current clinical efforts are focused on immune-based therapies, such as the inhibition of auto-reactive T cells, activation of regulatory T cells (Tregs), and regulation of B cell activity to prevent the relapse of this category of disorders (7, 8). However, it is important to note that the current understanding is limited as the study only examined a few commonly studied subsets of immune cells. The causal relationship between MS and peripheral immune cells remains unclear and requires further investigation.

Although the randomized controlled trial is widely regarded as the most reliable and rigorous method for generating clinical evidence, it can be challenging due to the need for a large sample size and significant human and financial resources. Mendelian randomization (MR) is an alternative approach that uses instrumental variables (IVs) assessed in exposure-related genome-wide association studies (GWAS) to evaluate the causal relationship between exposure and outcome, which needs to meet three key assumptions that IVs should be strongly related to exposure, independent of any confounding factors, and conditional independence with the outcome conditional on the exposure (9). Based on the random allocation of gametes during meiosis, MR has the ability to reduce confounding factors and strengthen causal inference (9). In this study, instrumental variables derived from large-scale genome-wide association studies (GWAS) of hematological traits were utilized to evaluate the causal relationship between peripheral immune cell counts and MS risk and to investigate their impact on MS severity using the MR approach.

## 2 Materials and methods

### 2.1 Study design

**Figure 1:**
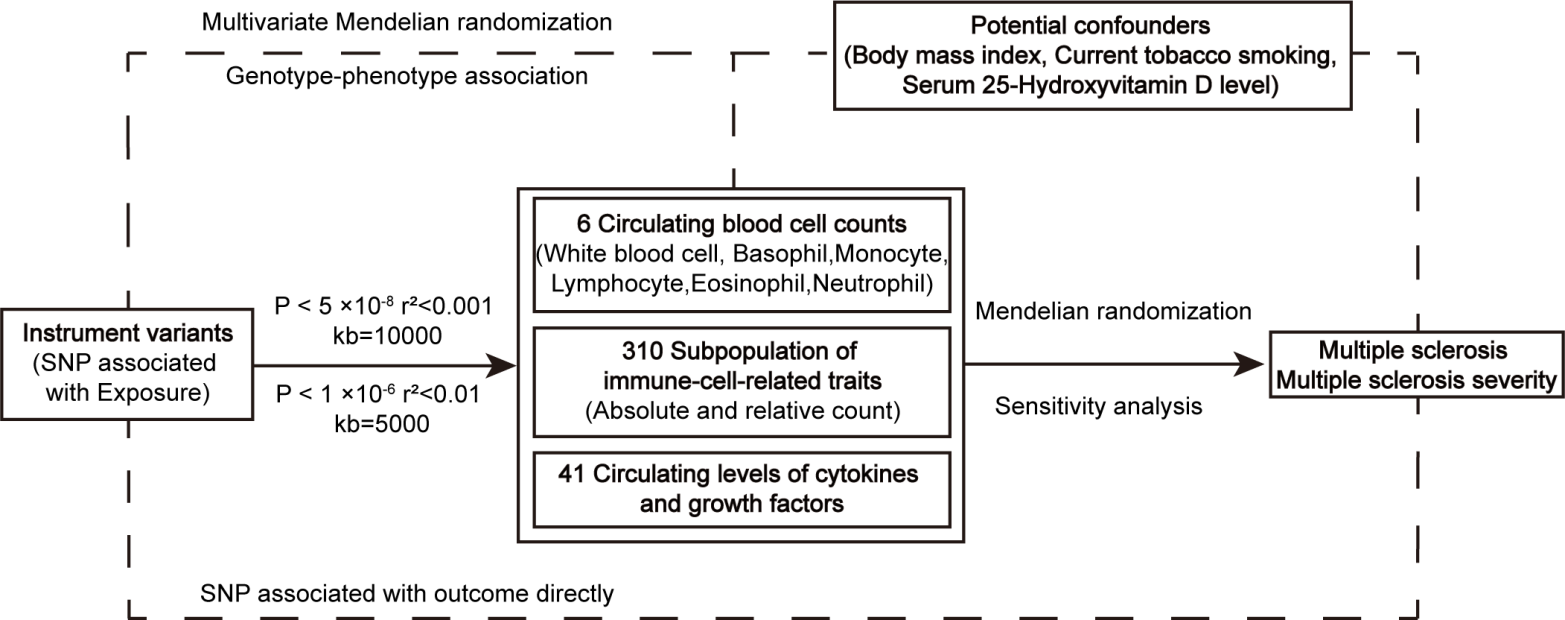
The workflow of instrumental SNP selection and Mendelian randomization analysis. SNP, single nucleotide polymorphism.

MR analysis was utilized to evaluate the causal relationship between exposure and outcome genetically. The effect of immune cell counts on the risk and disease severity of MS was measured using summary statistics from GWAS. Firstly, we aimed to investigate whether circulating cell counts in blood routine examination, including white blood cell, monocyte, neutrophil, eosinophil, basophil, and lymphocyte, have causal effects on the risk and disease severity. Additionally, we assessed causal associations between more specific blood immune-cell-related traits and MS and its severity. Furthermore, we explored the associations between genetically predicted circulating levels of cytokines and growth factors, which are regulators of inflammation, and MS and its severity, to determine whether these inflammatory regulators played a role in the development and progression of MS (**Figure 1**).

### 2.2 Data sources

GWAS of six items of blood routine examination were collected from 563,085 European ancestry participants, and obtained from UK Biobank cohort (N = 408,112) and Blood Cell Consortium participants (N = 154,973) (10). The absolute count and relative count of subpopulations of immune-cell-related traits originated from a cohort of 3,757 Sardinians, and the 310 peripheral immune cells phenotyping were divided into seven panels by flow cytometry, including TBNK panel, Treg panel, Maturation stages of T-cell panel, DC panel, B-cell panel, Monocyte panel and Myeloid cell panel (11). Single nucleotide polymorphisms (SNPs) associated with circulating levels of cytokines and growth factors were obtained from The Cardiovascular Risk in Young Finns Study and FINRISK, including 8,293 participants. And in this study, a total of 41 cytokines were measured by using Bio-Rad’s premixed Bio-Plex Pro Human Cytokine 27-plex Assay and 21-plex Assay, and Bio-Plex 200 reader with Bio-Plex 6.0 software (12). Body mass index (BMI), smoking, and serum vitamin D level were regarded as potential confounders, which were reported in previous studies (13–15).

International Multiple Sclerosis Genetics Consortium (IMSGC) used genetic samples from patients who have been diagnosed with MS by clinicians and professional medical teams to generate GWAS data of MS and its severity. Summary statistics data for MS included 115,803 European-descent individuals (47,429 cases and 68,374 controls) from the discovery and replication phases (16). The publicly available GWAS summary statistics for MS severity were obtained from 12,584 cases who had effectively declared their clinical outcome with a mean age at last follow-up and disease duration was 51.7 and 18.2 years, and the Expanded Disability Status Scale (EDSS) score was the indicator for neurological disability (17). GWAS data was available from public websites and labeled references (**Supplementary Table S1**).

### 2.3 Instrumental variable selection

For SNP of white blood cell count, monocyte count, neutrophil count, eosinophil count, basophil count, lymphocyte count, and potential confounders, 5 × 10^-8^ was selected as the threshold of P value to definite genome-wide significant SNP. We clumped the SNPs using a r^2^ < 0.001 clumping threshold and a clumping window of 10,000 kb to control the linkage imbalance. To purify the estimation of causal effect, SNPs that directly related to outcome were also removed (P < 1 × 10^-5^). Given the relatively moderate scale of sample size, P value threshold of 1 × 10^-6^, r^2^ < 0.01 within a clumping window of 5,000 kb, was used to filtrate SNPs associated with subgroups of immune cells, cytokines, and growth factors, and SNPs whose P < 1 × 10^-6^ in MS and its severity were removed to exclude a direct relationship with outcome. SNPs showing suggestive relationship (P < 1 × 10^-5^) with potential confounders were removed from MR analysis by retrieving a database of human genotype-phenotype associations (18). At last, F-statistics for each SNP were calculated and strong instrument variables with F > 10 remained. Variance explained (R^2^) for each genetic variant was calculated by 2×beta^2^×EAF×(1-EAF), and formula R^2^×(n-1-k)/k(1- R^2^) or beta^2^/se^2^ (when EAF was missing) was used to calculate F statistic, where beta, se was the estimated effect and standard error of the instrumental variable respectively, EAF was effect allele frequency, n represented sample size of exposure and k indicated the number of included variants.

### 2.4 Statistical analysis

Analyses were performed using the “TwoSampleMR” package, the “MendelianRandomization” package, and the “MRPRESSO” package in R software 4.2.2 (https://www.r-project.org/). We chose the Inverse variance weighted (IVW) method as the main method with the greatest statistical power, and Wald ratio was used when there was one SNP (19). MR Egger was implemented as a complementary method to assess the reliability of the IVW estimates. Then confounders were identified by analyzing the causal effect of common risk variables for MS on both exposure and outcomes, followed by multivariate Mendelian randomization to investigate the direct effects of blood cell count on MS risk and its severity, rather than act through the confounders (20). Sensitivity analysis included heterogeneity test and pleiotropy test. Cochran’s Q statistic was conducted to examine the heterogeneity and the MR-Egger method was conducted to examine pleiotropy. MR-PRESSO was further executed to recognize and eliminate outliers when pleiotropy existed for the number of SNP more than or equal to three (21). For the number of SNP less than three, associated traits for each SNP were reported through the PhenoScanner database for pleiotropy analysis. False discovery rate (FDR) correction was separately performed between six types of peripheral blood cell counts, 310 peripheral immune cell phenotypes, 41 circulating levels of cytokines, growth factors, and MS risk and its severity to control for the proportion of false positives in multiple testing. And a P adjust less than 0.05 after FDR would be regarded as a significant causal effect.

## 3 Results

### 3.1 Association of genetically predicted peripheral blood cell counts and the risk and severity of MS

**Figure 2:**
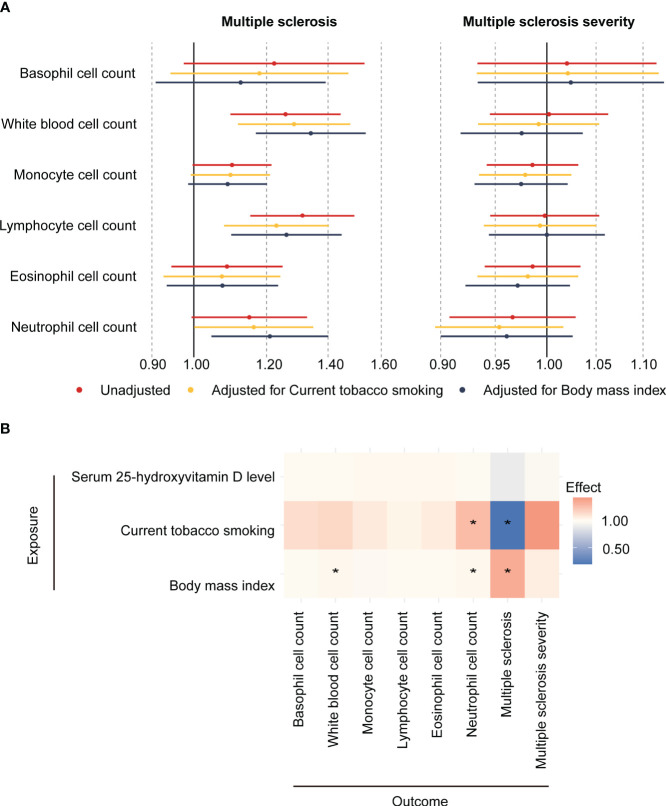
Association between circulating blood cell counts, confounders, and the risk and severity of MS. **(A)** Two-sample mendelian randomization and Multivariate mendelian randomization estimates of the association between peripheral cell counts and the risk of MS and its severity. **(B)** Two-sample Mendelian randomization estimates the causal effect of potential confounders on blood cell counts, multiple sclerosis, and multiple sclerosis severity. * means P < 0.05.

We first evaluated the relationships between confounding factors and both the exposures (peripheral blood cell counts) and outcomes (MS and MS severity). These findings, represented in **Figure 2B**, **Supplementary Table S2**, indicated that current tobacco smoking had significant associations with neutrophil cell count (β [95%CI] = 0.24 [0.09,0.39], P = 6.21E-03) and MS (OR [95%CI] = 0.19 [0.06, 0.63], P = 1.56E-02). Similarly, BMI was found to be related to white blood cell count (β [95%CI] = 0.03 [0.00,0.05], P = 1.64E-02), neutrophil cell count (β [95%CI] = 0.02 [0.00,0.04], P = 4.65E-02) and MS (OR [95%CI] = 1.34 [1.17,1.54], P = 3.78E-05).

In the primary MR analysis, it was found that genetically determined higher white blood cell count (OR [95%CI] = 1.26 [1.10,1.44], P = 1.12E-03, P adjust = 3.35E-03) and lymphocyte cell count (OR [95%CI] = 1.31 [1.15,1.50], P = 5.37E-05, P adjust = 3.22E-04) increased the risk of MS. To correct the influence of confounding factors, multivariate Mendelian randomization was performed, adjusting for current tobacco smoking and body mass index. In model 1 (adjusted for current tobacco smoking), higher white blood count (OR [95%CI] = 1.29 [1.12, 1.48], P = 4.69E-04, P adjust = 2.81E-03) and lymphocyte cell count (OR [95%CI] = 1.23 [1.08, 1.40], P = 2.00E-03, P adjust = 6.00E-03) increased the risk of MS. In model 2 (adjusted for BMI), higher white blood cell count (OR [95%CI] = 1.34 [1.17, 1.54], P = 3.02E-05, P adjust = 1.81E-04), lymphocyte cell count (OR [95%CI] = 1.26 [1.10, 1.45], P = 1.01E-03, P adjust = 3.02E-03) and neutrophil cell count (OR [95%CI] = 1.21 [1.05, 1.40], P = 1.07E-02, P adjust = 2.15E-02) increased in risk of MS. Among them, the estimated effects of IVW and MR-Egger were in a consistent direction. However, no association was found between these blood cell counts and the severity of MS (**Figure 2A**; **Supplementary Table S3**). This suggests that while blood cell count and lymphocyte cell count may influence the risk of developing MS, they may not directly impact the severity of the disease.

### 3.2 Causal effect of immune cell subtypes on the risk and severity of MS

**Figure 3:**
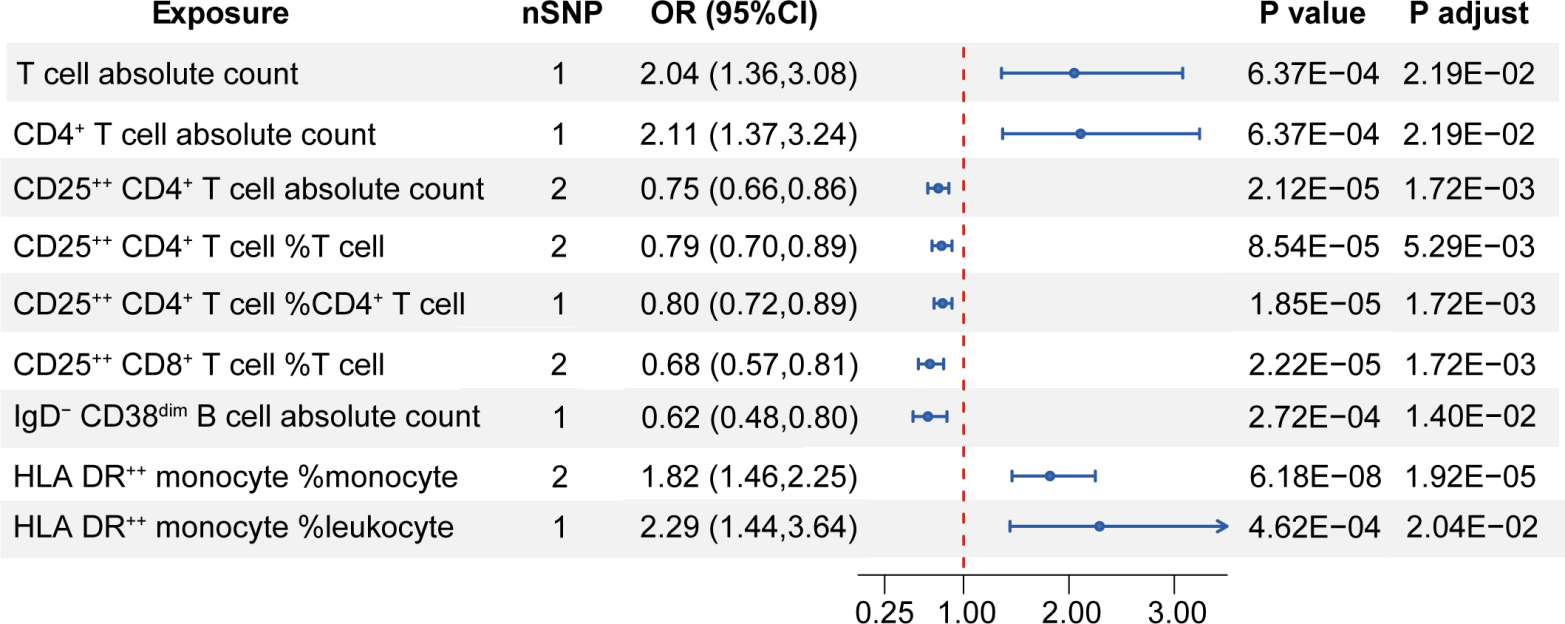
Mendelian randomization estimates of the relationship between the subgroup of immune cells and MS. nSNP, the number of single nucleotide polymorphism; OR, odds ratio; CI, confidence interval; P adjust, P value adjusted by the false discovery rate method. Only significant results with P adjust < 0.05 after FDR adjustment are shown.

For a more detailed subgroup of immune cells, nine immune-cell-related traits demonstrated causal effect on the risk of MS. A higher absolute count of total T cell (OR [95%CI] = 2.04 [1.36,3.08], P = 6.37E-04, P adjust = 2.19E-02) and CD4^+^ T cell (OR [95%CI] = 2.11 [1.37,3.24], P = 6.37E-04, P adjust = 2.19E-02) increased the risk of MS. Interestingly, it was observed that a higher expression of CD25 on T cell decreased in MS risk. Genetically predicted higher CD25^++^CD4^+^ T cell absolute count showed a reduction in MS susceptibility (OR [95%CI] = 0.75 [0.66,0.86], P = 2.12E-05, P adjust = 1.72E-03), as well as the proportion of CD25^++^CD4^+^ T cell in T cell (OR [95%CI] = 0.79[0.70,0.89], P = 8.54E-05, P adjust = 5.29E-03) and in CD4^+^ T cell (OR [95%CI] = 0.80[0.72,0.89], P = 1.85E-05, P adjust = 1.72E-03). In addition, higher relative count of CD25^++^CD8^+^ T cell in T cell was also found to decrease the risk of MS (OR [95%CI] = 0.68[0.57,0.81], P = 2.22E-05, P adjust = 1.72E-03). Furthermore, we revealed that higher proportion of HLA DR^++^ monocyte of total monocyte (OR [95%CI] = 1.82[1.46,2.25], P = 6.18E-08, P adjust = 1.92E-05) and of total leukocyte (OR [95%CI] = 2.29[1.44,3.64], P = 4.62E-04, P adjust = 2.04E-02) increased in MS risk. On the other hand, higher IgD^-^CD38^dim^ B cell count was found to decrease the risk of MS (OR [95%CI] = 0.62[0.48,0.80], P = 2.72E-04, P adjust = 1.40E-02) (**Figure 3**). Results of the heterogeneity test indicated no heterogeneity (P values for Cochran’s Q > 0.05) existing among these selected instruments and pleiotropy analysis for each SNP found no traits associated with MS obviously. High statistical power was shown, and we validated our significant results with different MR analysis methods, which exhibited high stability (**Supplementary Table S5**).

Regarding the severity of MS, some immune-cell-related traits showed a suggestive causal association (P < 0.05 while P adjust > 0.05). Increased level of resting Treg cell count (OR [95%CI] = 0.96[0.92,0.99], P = 2.28E-02, P adjust = 9.51E-01), proportion of resting Treg in CD4^+^ Treg (OR [95%CI] = 0.98[0.96,1.00], P = 4.55E-02, P adjust = 9.51E-01), proportion of naïve CD4^+^ T cell in CD4^+^ T cell (OR [95%CI] = 0.93[0.88,0.98], P = 1.06E-02, P adjust = 9.51E-01), proportion of terminally differentiated CD4^+^ T cell in CD4^+^ T cell (OR [95%CI] = 0.94[0.89,0.99], P = 2.11E-02, P adjust = 9.51E-01), and proportion of CD25^++^CD8^+^ T cell in CD8^+^ T cell (OR [95%CI] = 0.94 [0.89,0.99], P = 1.73E-02, P adjust = 9.51E-01) decrease the severity of MS. Conversely, higher white blood cell count (OR [95%CI] = 1.06[1.00,1.12], P = 4.09E-02, P adjust = 9.51E-01), higher HLA DR^++^ monocyte count (OR [95%CI] = 1.13[1.00,1.28], P = 4.21E-02, P adjust = 9.51E-01), higher CD66b^++^ myeloid cell count (OR [95%CI] = 1.12[1.02,1.23], P = 2.15E-02, P adjust = 9.51E-01), higher proportion of activated and secreting Treg in CD4^+^ Treg (OR [95%CI] = 1.02[1.00,1.04], P = 1.51E-02, P adjust = 9.51E-01), higher proportion of effect memory CD4^+^ Treg in T cell (OR [95%CI] = 1.04[1.00,1.09], P = 3.94E-02, P adjust = 9.51E-01), and higher proportion of IgD^+^CD24^-^ B cell in B cell (OR [95%CI] = 1.15[1.01,1.31], P = 3.32E-02, P adjust = 9.51E-01) were associated with increasing MS severity (**Supplementary Table S6**). And the estimated effects of IVW and MR-Egger were shown consistent direction. Sensitivity analyses suggested no heterogeneity and no obvious bias from genetic pleiotropy.

### 3.3 Causal effect of cytokines and growth factors on the risk and severity of MS

**Figure 4:**
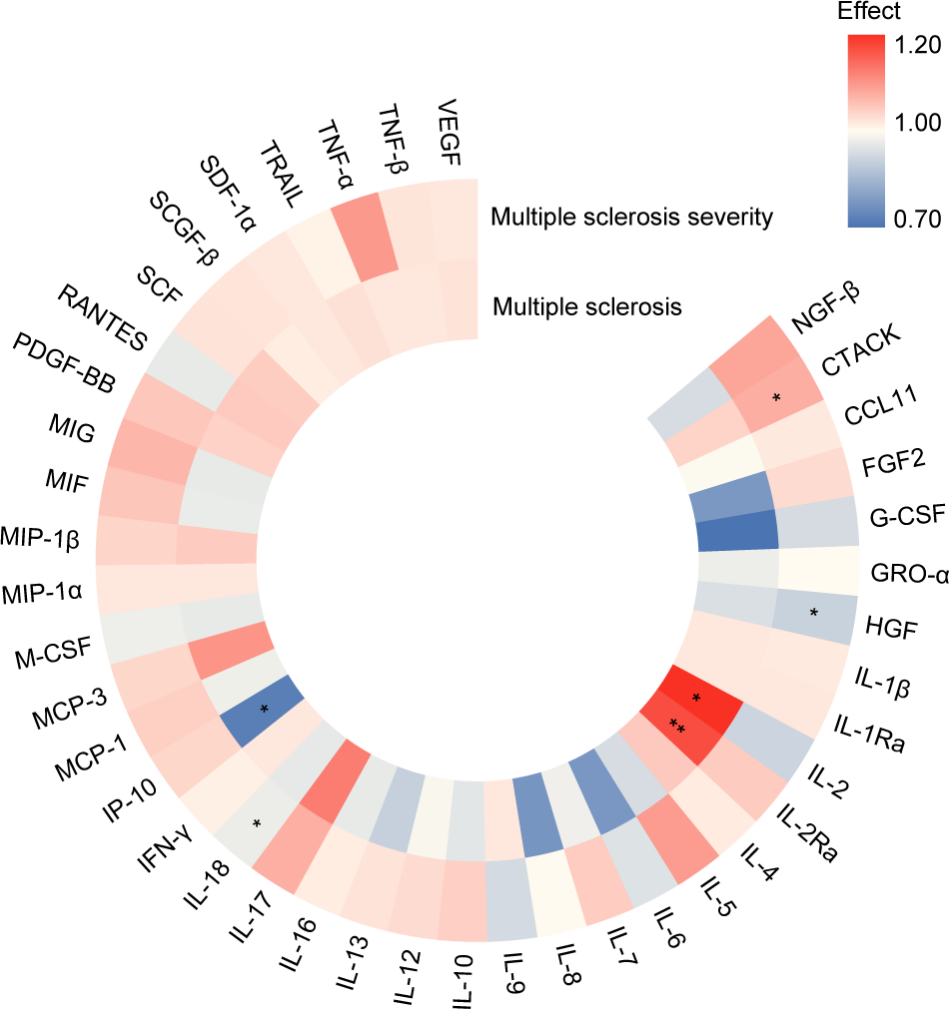
Summary of associations of genetically predicted levels of cytokines and growth factors with risk of MS and its severity through Mendelian randomization. Color gradient represents the effect estimation of a causal relationship (red: detrimental factors, blue: protective factors), * means P < 0.05, ** means P adjust < 0.05. (https://www.chiplot.online/).

When evaluating the relationship between cytokines and growth factors and MS and its severity, we found that higher level of IL-2Ra was associated with increasing risk in MS (OR [95%CI] = 1.22 [1.12,1.32], P = 3.20E-06, P adjust = 1.34E-04). On the other hand, higher level of IP-10 (OR [95%CI] = 0.71 [0.55,0.91], P = 7.72E-03, P adjust = 1.62E-01) decreased the risk of MS, and higher level of IL-2 (OR [95%CI] = 1.26 [1.00,1.57], P = 4.61E-02, P adjust = 6.46E-01) increased the risk of MS with suggestive evidence (P < 0.05). In terms of MS severity, a higher level of CTACK increased MS severity (OR [95%CI] = 1.08 [1.02,1.15], P = 9.96E-03, P adjust = 3.14E-01). Conversely, higher levels of IL-18 (OR [95%CI] = 0.94 [0.90,0.99], P = 1.53E-02, P adjust = 3.14E-01) and HGF (OR [95%CI] = 0.89 [0.79,1.00], P = 4.73E-02, P adjust = 5.82E-01) decreased MS severity with suggestive evidence (**Figure 4**; **Supplementary Tables S7**, **S8**).

## 4 Discussion

In recent years, GWAS have greatly advanced our understanding of the genetic factors involved in MS, with over 200 associated loci identified (16). Although the precise mechanism is complex and not fully understood, there is increasing evidence suggesting that the immune response, including immune cell recruitment, activation, and cytokine release, plays a role in the progression of MS (22, 23). In this study, a causal relationship between peripheral immune cell counts and the risk of MS, as well as MS severity, was assessed through the MR approach. We revealed that peripheral white blood cell count and lymphocyte count were closely associated with the risk of MS. These findings align with previous research indicating that MS patients often exhibit elevated levels of these immune cells (24, 25). Meanwhile, current studies reveal that the ratios between peripheral immune cells, such as neutrophil-lymphocyte ratio (NLR) and monocyte-lymphocyte ratio (MLR), are robustly associated with MS severity (26) and risk of MS recurrence within two years (27). Elevated NLR at the outset of relapsing-remitting multiple sclerosis (RRMS) is linked to disease activity (28), suggesting that it could be a reliable inflammatory biomarker for predicting disease activity in MS.

Considering that the overall effects can be different based on specific cell subtypes, our study further investigated immune cell subsets. It identified five subtypes of immune cells that were protective against MS and four that were detrimental. Specifically, higher absolute counts of total T cell and CD4^+^ T cell were found to have a detrimental effect on MS. This could be attributed to the persistent migration of autoreactive T cells and other inflammatory immune cells from peripheral regions into the brain and spinal cord, resulting in demyelination of the neural axons (29). In normal immune responses, CD4^+^ T cells show significant plasticity in terms of differentiation (30). In MS, there is an expansion of Th1/Thl7 cells, a decrease in Th2/Treg cells, and elevated levels of interferon γ levels (31). The primary drive of autoimmunity is Th17 cells, which have a stronger proliferation ability than Th1 cells. Additionally, Th17 cells inhibit the differentiation of other lymphocyte subtypes, particularly through the production of cytokines such as transforming growth factor beta (TGF-β) (32). Given that there are many subsets of T cells, subsequent further discussions of their roles are necessary.

In the study, CD25^++^CD4^+^ T cells, a subset of CD4^+^T cells, were also demonstrated to reduce MS susceptibility. CD25 is an interleukin (IL)-2 receptor α as a surface marker for Tregs. Tregs play a crucial role in maintaining immune homeostasis, avoiding autoimmune disorders, and minimizing transplant rejection, by the suppression of the immune response of various cell types, including effector CD4^+^ T cells (33). Tregs exert their inhibitory effects on effector T cells through direct binding to specific receptors on target cells, such as cytotoxic T lymphocyte-associated antigen 4 (CTLA-4), TGF-β, and anti-glucocorticoid-induced tumor necrosis factor receptor (GITR) (34, 35). As a result, effector T cells exhibit reduced responsiveness to IL-2 due to decreased expression of the IL-2 receptor chain (36). In MS patients, Tregs are deficient or defective in the blood, brains, and cerebrospinal fluid (CSF), characterized by reduced expression of the Forkhead box P3 (Foxp3) protein (37), which impairs their ability to adequately suppress the immune response of T cells towards the myelin basic protein (38). Interferon-β, the preferred immunomodulatory drug for RRMS, increases the frequency of Tregs and restores their inhibitory function by upregulating Foxp3 expression levels (39). Our MR research corroborated the causative relationship between CD4^+^ Tregs and MS, supporting the point that individuals with more CD4^+^ Tregs are less likely to suffer from MS. Our study provided suggestive evidence that the resting Tregs and a higher proportion of resting Tregs in CD4^+^ Tregs might decrease the MS severity though they cannot pass the FDR adjustment. And an observational study indicated that the frequency of Tregs in MS patients reduced compared with healthy controls, and there is a negative connection between Tregs frequency and EDSS in MS (40). Therefore, it is necessary to further study the possibility of Tregs becoming a target for alleviating the severity of MS.

In addition to the natural CD4^+^ Tregs, another class of induced Tregs, including CD8^+^ Tregs, deserves attention. Though the phenotypic characteristics of CD8^+^ Tregs resemble those of naturally generated CD4^+^ Tregs, featuring higher levels of Foxp3, GITR, and CTLA-4, and reduced levels of CD127 (41), the functionality of CD4^+^ Tregs has been extensively researched, while there has been relatively little attention given to the research of CD8^+^ Tregs. Our study also found that a higher percentage of CD25^++^CD8^br^ T cell in T cell and a higher percentage of CD25^++^CD8^br^ T cell in CD8^br^ T cell decreased the risk of MS and have the potential to alleviate MS severity, respectively. According to a previous study, CD8^+^ Tregs are less frequent in the blood and show impaired function in MS patients (42). In MS and other experimental autoimmune disorders, researchers have discovered the beneficial effect of T cell vaccination, which stimulates the occurrence of CD8^+^ Tregs, and results in the selective downregulation of self-reactive CD4^+^ T cells (41). Given the inhibitory effect of CD8^+^ Tregs on self-response, it may become a candidate for future intervention in the occurrence and development of autoimmune diseases.

Additionally, IgD^-^CD38^dim^ B cells, belonging to Switched Memory B cells, were discovered to decrease the risk in MS. Class-switched memory B cells had a higher frequency in the CSF than in the blood sample, suggesting that class-switched memory B cells may divert from the periphery into the CSF region (43). Another B cell subpopulations-IgD^+^CD38^-^ B cells (Unswitched Memory B cells) decreased in RRMS patients when compared with healthy controls (44). Unswitched B memory cells, defined as mainly virgin naive cells, are highly reactive and phenotypically comparable to Switched Memory B cells (45). The study has shown that RRMS and secondary progressive multiple sclerosis have higher levels of memory B cells in their peripheral blood (46). When stimulated by B cell receptor and CD40, memory B cells in MS patients can effectively present neuronal antigens to T cells, activate T cells, and secrete Tumor necrosis factor-α (47). The administration of Atacicept, which enhances memory B cell responses, has been observed to promote MS recurrence, indicating the significant involvement of memory B cells in MS relapse (48). Cladribine, which potentially depletes memory B cells, has been approved for the treatment of relapsing MS (49). Therefore, further research should be devoted to investigating the potential pathways of how IgD^-^CD38^dim^ B cells impact MS, offering prospective therapeutic avenues for clinical application.

Furtherly, we demonstrated that the proportion of HLA DR^++^ monocyte of total monocyte and of total leukocyte increased the risk of MS, and HLA DR^++^ monocyte might be associated with higher severity of MS. HLA-DR represents a class II major histocompatibility complex antigen that is prominently present on the outer membrane of B lymphocytes, monocytes, and macrophages. Its primary role is to facilitate the process of antigen presentation to CD4^+^ T cells. Previous studies have identified HLA-DRB1*15:01 as a significant genetic factor contributing to the development of MS, increasing the risk by threefold (50). It is worth mentioning that monocytes play anti-inflammatory and pro-inflammatory functions in the early phases of the disease (51), suggesting the necessity of investigating the function of multiple monocyte subtypes in MS onset. And in our study, CD14^+^CD16^+^ monocyte, which represents the pro-inflammation phenotype, and the proportion of CD14^+^CD16^+^ monocyte in monocyte showed a suggestive causal association with increased risk in MS. Current study has shown that patients with RRMS have a higher frequency of CD14^+^CD16^+^ monocyte compared to healthy controls (44), which is consistent with our study and provide its potential as a biomarkers for MS. Moreover, the effect of myeloid-derived suppressor cells (MDSCs) in regulating the immune response, as well as its immunosuppressive properties in autoimmune diseases, prompt that its role in MS cannot be ignored. In the experimental autoimmune encephalomyelitis (EAE) model, MDSCs can trigger T cell apoptosis to suppress inflammation and boost recovery of EAE symptoms (52). Similarly, the abundance of Monocytic MDSC in blood samples from untreated MS patients at the time of first relapse had a negative correlation with the EDSS at baseline and after one-year follow-up (53), underscoring the need for dynamic MDSC monitoring in the development of MS.

Finally, we have discovered that interleukin-2 receptor antagonist (IL-2Ra) increased susceptibility to MS. IL-2 is known to enhance the survival and proliferation of activated T cells, as well as the differentiation of effector T cells, by binding to the IL-2 receptor (54). IL-2Ra blocks the downstream signaling of the IL-2 receptor by specifically antagonizing its α chain (55). However, previous studies have shown that IL-2Ra has the capacity to form complexes with IL-2, such as sIL-6R-IL-6 (56) and sIL-15R-IL-15 complexes (57), thereby enhancing signaling. It should be noted, though, that this occurrence is less likely. Therefore, further in-depth and comprehensive research is required to explore the exact role of immune factors on MS.

The reliability of our research findings was strengthened by the utilization of recently published high-quality GWAS. The study was designed meticulously with reliable data sources utilized, resulting in a substantial level of statistical power. However, it is necessary to acknowledge the limitations of the MR method discussed in the literature review. Our study has some limitations as follows: firstly, the research primarily focuses on participants of European ancestry. Therefore, the research results cannot be generalized to other ethnic groups. It is essential to replicate the study in other populations. Secondly, analyzing immune cell subtypes based on small sample sizes may result in insufficient ability to detect the impact of immune cell subtypes on the risk and severity of MS. Thirdly, various transient variables, such as age and lifestyle factors, could influence an individual’s immunological characteristics at a given moment, which may not correspond to the immune features defined by coding genes throughout an individual’s lifetime.

## 5 Conclusion

In conclusion, our study has identified the causal relationship between peripheral immunity and MS through Mendelian randomization. These findings have the potential to serve as biomarkers for predicting the onset and development of MS and offer new insights into the underlying mechanisms involved in MS. Further research is needed to better understand the specific relationship between peripheral immune cells and MS.

## Data availability statement

The original contributions presented in the study are included in the article/**Supplementary Material**. Further inquiries can be directed to the corresponding authors.

## Author contributions

LC: Conceptualization, Data curation, Formal analysis, Investigation, Methodology, Software, Validation, Visualization, Writing – original draft. L-FZ: Conceptualization, Data curation, Formal analysis, Investigation, Methodology, Software, Validation, Visualization, Writing – original draft. L-YZ: Conceptualization, Data curation, Formal analysis, Investigation, Methodology, Writing – original draft. Y-HC: Conceptualization, Data curation, Formal analysis, Investigation, Methodology, Writing – original draft. M-HD: Formal analysis, Investigation, Methodology, Writing – original draft. X-WP: Formal analysis, Investigation, Methodology, Writing – original draft. SY: Software, Validation, Visualization, Writing – original draft. L-QZ: Software, Validation, Visualization, Writing – original draft. KS: Software, Validation, Visualization, Writing – review & editing. JX: Software, Validation, Visualization, Writing – review & editing. WW: Funding acquisition, Writing – review & editing. CQ: Conceptualization, Data curation, Funding acquisition, Writing – review & editing. D-ST: Conceptualization, Data curation, Funding acquisition, Writing – review & editing.
